# Current challenges and practical aspects of molecular pathology for non-small cell lung cancers

**DOI:** 10.1007/s00428-023-03651-1

**Published:** 2023-10-06

**Authors:** Paul Hofman, Sabina Berezowska, Daniel Kazdal, Baharia Mograbi, Marius Ilié, Albrecht Stenzinger, Véronique Hofman

**Affiliations:** 1 Côte d’Azur University, FHU OncoAge, IHU RespirERA, Laboratory of Clinical and Experimental Pathology, BB-0033-00025, Louis Pasteur Hospital, 30 avenue de la voie romaine, BP69, 06001 Nice cedex 01, France; 2 Côte d’Azur University, IRCAN, Inserm, CNRS 7284, U1081 Nice, France; 3 Department of Laboratory Medicine and Pathology, Institute of Pathology, Lausanne University Hospital and University of Lausanne, Lausanne, Switzerland; 4 Translational Lung Research Center Heidelberg (TLRC), German Center for Lung Research (DZL), Heidelberg, Germany; 5 Centers for Personalized Medicine (ZPM), Heidelberg, Germany; 6 Institute of Pathology, Heidelberg University Hospital, Heidelberg, Germany

**Keywords:** Lung cancer, Molecular pathology, Predictive biomarkers, Targeted therapy, Immunotherapy

## Abstract

The continuing evolution of treatment options in thoracic oncology requires the pathologist to regularly update diagnostic algorithms for management of tumor samples. It is essential to decide on the best way to use tissue biopsies, cytological samples, as well as liquid biopsies to identify the different mandatory predictive biomarkers of lung cancers in a short turnaround time. However, biological resources and laboratory member workforce are limited and may be not sufficient for the increased complexity of molecular pathological analyses and for complementary translational research development. In this context, the surgical pathologist is the only one who makes the decisions whether or not to send specimens to immunohistochemical and molecular pathology platforms. Moreover, the pathologist can rapidly contact the oncologist to obtain a new tissue biopsy and/or a liquid biopsy if he/she considers that the biological material is not sufficient in quantity or quality for assessment of predictive biomarkers. Inadequate control of algorithms and sampling workflow may lead to false negative, inconclusive, and incomplete findings, resulting in inappropriate choice of therapeutic strategy and potentially poor outcome for patients. International guidelines for lung cancer treatment are based on the results of the expression of different proteins and on genomic alterations. These guidelines have been established taking into consideration the best practices to be set up in clinical and molecular pathology laboratories. This review addresses the current predictive biomarkers and algorithms for use in thoracic oncology molecular pathology as well as the central role of the pathologist, notably in the molecular tumor board and her/his participation in the treatment decision-making. The perspectives in this setting will be discussed.

## Introduction

The progress in the discovery of different therapeutic targets in combination with immunotherapy has substantially improved the survival of patients with non-small cell lung cancer (NSCLC), particularly non-squamous (NS)-NSCLC at advanced and even at early disease stages [1, 2]. Based on international recommendations, targeted therapies can only be administered if the molecular targets have been identified [3, 4]. They include genomic alterations (mutations, amplifications, rearrangements) or changes in protein expression [5, 6]. Due to the continuing results of translational research and clinical trials, the list of predictive biomarkers which require analysis, is progressively growing [7, 8]. Thus, the identification of novel actionable molecular targets or those associated with therapeutic resistance will lead to a wide scope of molecular alterations to be identified in daily practice [9, 10]. The identification of these targets is performed in clinical and molecular pathology laboratories.

The responsibility of pathologists in thoracic oncology has grown steadily in recent years. They now participate in multidisciplinary/molecular tumor boards (MTB) in which they play an essential role in determining therapeutic strategies [11]. During the histological diagnostic process, besides to be strongly involved in the molecular biology analyses and interpretation, one of the major concerns of the pathologist is to evaluate the quality and quantity of the biological sample used for the subsequent analyses of predictive biomarkers. The need to assess the percentage of tumor cells and the area of necrosis, to prescribe immunohistochemical (IHC) and molecular analyses and to decide on the methods of choice [e.g., targeted molecular analyses by RT-PCR, In-Situ Hybridization (ISH), Next-Generation Sequencing (NGS)] has turned the pathologist into a major actor in thoracic oncology workflow, and, thus a key participant in therapeutic decision-making. The pathologist is also responsible for controlling the delay of transmitting the required diagnostic, IHC, and molecular results for administration of appropriate therapeutic strategies. With this in mind, the pathologist must also request additional tissue and/or liquid biopsy sample material from the clinician, if the amount of tissue or cytological material is insufficient for a complete analysis of the requested biomarkers. Depending on the type of organization and the level of expertise of the laboratory, the respective molecular analyses including tissue and/or liquid material obtained at diagnosis and/or at progression of the tumor are performed at the pathology laboratory. Ideally, the pathologist should participate in the interpretation of both the morphological and molecular results. However, the molecular biologists have a pivotal role in the assessment of the different genomic alterations, notably of some complex genomic aberrations, and for integrating for example different information concerning the genetic risks for cancer predisposition. Therefore, according to the hospital organization, many of them are part of the pathology department and are working hand in hand with the pathologists.

This review will examine primarily the molecular biomarkers that must be evaluated in the daily practice of thoracic oncology, but also emerging biomarkers under investigation. Then, the role of the pathologist in the management of tests in molecular biology in thoracic oncology, and the potential difficulties, will be discussed. Finally, some perspectives will be considered.

## Molecular biomarkers for lung cancers

Predictive biomarkers in thoracic oncology can be broadly divided into three categories according to the currently recognized level of response to therapeutics associated with their detection: (1) routinely detected biomarkers, (2) emerging biomarkers, and (3) biomarkers under investigation (Table 1).

**Table 1:**
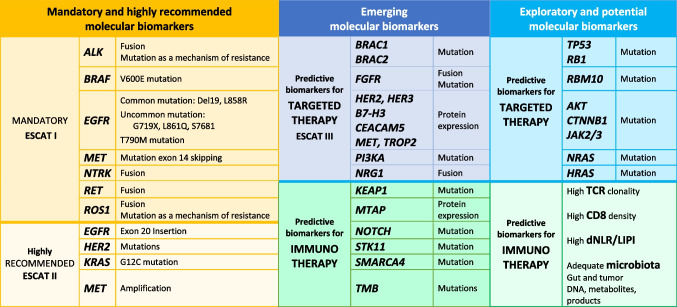
Mandatory, emerging, and exploratory predictive biomarkers for targeted therapies and immunotherapies in non-small cell lung cancers

### Molecular biomarkers evaluated in daily routine

International recommendations provide a list of predictive biomarkers to be identified in thoracic oncology [12–14]. It is necessary to adhere to this list to make therapeutic state-of-the-art decisions for patients with NS-NSCLC (Table 1). Most of these biomarkers are presently looked for using tissue or cellular samples of advanced or metastatic tumors [14]. They are associated with molecular therapeutics approved by the Food and Drug Administration (FDA) and/or the European Medical Agency (EMA). Detection of those biomarkers is a prerequisite to the administration of therapeutic molecules that improve survival of patients. These biomarkers are associated with actionable targets and are classified into category I (A, B, C) by the European Society for Medical Oncology (ESMO) according to the ESCAT classification (ESMO Scale for clinical actionability of molecular targets) [14]. Some of these molecules are administered as first-line treatments in stage IV disease. They target actionable mutations in *EGFR* and rearrangements in *ALK* and *ROS1* [15]. Other molecular therapeutics are administered as second-line treatment after tumor progression on chemotherapy and/or immunotherapy, but some of them are now administered as first-line treatment in some countries thanks to their reimbursement [12, 13]. They target *NTRK* rearrangements, acquired T790M mutations in exon 20 of *EGFR*, exon 20 insertions of *EGFR*, and other uncommon *EGFR* mutations (e.g., G719X in exon 18, L861Q in exon 21, S768I in exon 20), *MET* exon 14 skipping, *RET* rearrangements, and *BRAF* V600 mutations [12–14, 16–19]. Recent data concerning some targeted therapies provided clinical benefit to the patients and associated biomarkers have to be evaluated systematically [13]. Therefore, due to the recent FDA/EMA approval for the administration of sotorasib and of amivantamab for targeting *KRAS* G12C mutation and insertions in exon 20 of *EGFR*, respectively, these latter genomic alterations have to be considered mandatory to look for now [13, 18, 19]. These biomarkers can also be detected with liquid biopsies, in particular in blood samples but also in pleural and cerebrospinal fluids, but with variable sensitivity of detection [20, 21]. The investigation of these biomarkers must be performed concomitantly with the evaluation of the expression of PD-L1 on tumor cells by IHC. Moreover, the evaluation of the *EGFR* status in early-stage NS-NSCLC (stage IB-IIIA) is now recommended due to the recently approved adjuvant treatment options [22].

### Emerging biomarkers

Some biomarkers are not evaluated systematically in daily routine yet, but are recommended (Table 1) [14]. Therefore, some of these biomarkers are associated to clinical trials using molecules in second-line treatment. These biomarkers belong to category III of the ESCAT classification but their detection may soon become mandatory routinely for administration of novel therapeutic targets (Table 1) [14]. They concern fusions in *NRG1*, mutations in *PIK3CA*, *BRAC1/2*, *MAP2K1*, and *BRAF* non-V600, and amplifications in *HER2* [14, 23]. Moreover, some emerging biomarkers target genomic alterations in squamous NSCLC. Those are mostly fusions, mutations, and amplifications in *FGFR* [24].

In thoracic oncology, the development of antibody drug conjugates (ADCs) has led to new treatment options for NSCLC and small-cell lung cancer [25, 29], with associated predictive biomarkers [25–29]. These biomarkers (such as c-MET, TROP2, CEACAM5, DLL3, HER3, HER2, and B7-H3) can be identified using IHC or molecular tests [27, 30, 31].

Other biomarkers are being evaluated in the context of prediction of primary resistance to immune check point inhibitors (ICIs). They concern mutations in *STK11*, *KEAP1*, *SMARCA4*, or *NOTCH* or the loss of expression of MTAP [10, 32–35]. Finally, several predictive biomarkers of primary resistance to targeted therapies, notably tyrosine kinase inhibitors (TKI), are being studied [36, 37]. In this context, the analysis by NGS of all of the co-mutations associated to actionable mutations, notably in *EGFR* mutated tumors, can allow investigation into other biomarkers of resistance and partial response to targeted therapies, such as mutations in *P53* [37].

Finally, concerning the tumor mutation burden (TMB), different studies have shown controversial results. Conversely to some approval in the USA, it is not currently recommended at least in Europe to investigate TMB as a biomarker of response to immunotherapy in daily practice, but this could be of interest in the future [23, 38].

### Exploratory biomarkers

Preclinical studies (cellular and/or animal models) have shown the efficacy of molecules that may be used for treatment and that are often aimed at currently only hypothetical targets [39, 40]. However, no or insufficient clinical data is as yet available concerning the presence and clinical importance of those biomarkers.

### International recommendations published in 2023 concerning molecular testing with tissue and liquid biopsies for lung cancer diagnosis

In addition to individual national guidelines, there are a number of international organizations providing and regularly updating a list of mandatory biomarkers to assess for targeted therapies in thoracic oncology [14, 21, 41, 42]. Comprehensive recommendations for the analysis of tissue and liquid biopsy samples from a Europe perspective are given by the ESMO (European Society of Medical Oncology) [14, 20, 43]. The application of NGS is generally recommended for profiling molecular targets that are classified according to their actionability level based on well-established biomarkers that are recognized by the FDA and/or EMA but also on a number of emerging biomarkers [14]. The constant evolution of guidelines and recommendations regarding predictive biomarkers should raise concern as these biomarkers may undergo reclassification years after they are initially reported.

## Challenges faced by molecular pathologists in thoracic oncology: major concerns and perspectives

**Fig. 1:**
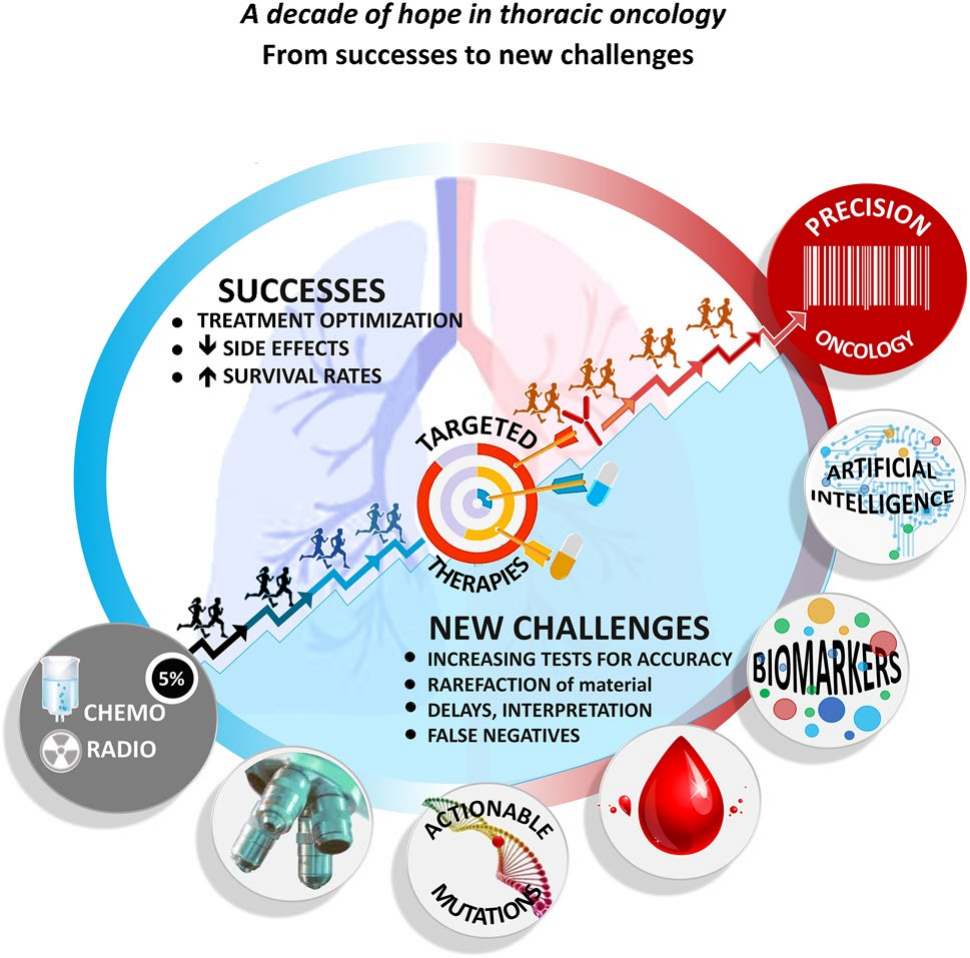
Progresses and challenges in thoracic oncology for molecular pathology

Over the past decade, therapeutic options for NSCLC patients have substantially improved largely attributed to precision oncology and the identification of predictive biomarkers. Despite these advancements, molecular pathologists still encounter challenges in conducting molecular testing (Figure 1) (Table 2).

**Table 2:** Issues for the thoracic oncologist for in-house molecular pathology setting

*Pre-analytical challenges*
Standardization of the sample workflow according to accreditation (ISO) processes
Mastering transport of samples and traceability up to sample registration in the laboratory
Best practices for sample storage (FFPE, slides, plasma, DNA/RNA)
Selection of samples for morphological/IHC analyses and for molecular testing
Decision making for NGS
Decision making for liquid biopsy request
Nucleic acids isolation (automation) and quality/quantity assessment
Clinical data set availability
*Analytical issues*
Selection of the technologies for molecular biology (single gene sequencing and NGS)
Selection of the NGS panel (s): small (< 50 genes) and/or medium (50 genes) and/or large (> 300 genes) panels
*Post analytical issues*
Interpretation and reporting of genomic alterations (standardization of the report)
Set up a comprehensive description and interpretation of the mutational profile of the assessed biomarkers
Informatics for report transmission
Follow the international recommendation in respecting the turnaround times to submit the report
*Accreditation and external quality assessment*
Accreditation of molecular biology testing from both tissue and fluid samples
Selection of and maintaining recognized national of international programs for external quality assessment
Anticipate issues associated with the IVDR
*Management of the laboratory*
Specific training for the laboratory staff
Promote as much as possible the automation of the pre-analytical and analytical phases
Reinforce the capacity of storage for genomic data
Possibility to increase the number of staff members
*Interaction with the physicians*
Establish continued dialogue for improvement of processing
Collective interpretation of the results/report
Mandatory participation in the multidisciplinary tumor board
*Convince the administration and the financial bodies*
Anticipate the activity, cost, reimbursement of molecular pathology testing at the local and national level
*Set up educational training*
Develop master classes for academic and private partners to:
Harmonize routine clinical practices and transmission of guidelines
Provide practical training on site

### Set up of the pre-analytical phase

The quality of the samples (tissue, cytological, or liquid samples) depends on the pre-analytical workup from sampling to morphological and molecular analysis. Effective management of this phase requires seamless communication and collaboration among clinical, surgical, and laboratory teams. Achieving and upholding accreditation according to the ISO 15189 standard necessitates the establishment of this essential point [44]. The different steps of the pre-analytical phase and their impact on diagnosis, in particular in molecular pathology, have already been widely described [45–47]. The pathologist plays an important role in the control of the handling of samples. Optimal handling of samples must be set up in collaboration with the pulmonologist and thoracic surgeon. In the laboratory, the time of fixation (e.g., 4% buffered formalin), the steps associated to embedding the tissue in paraffin sections, the amount of the tissue sections, the staining processes, and the tumor cell content can all have an impact on the quality of the results concerning predictive biomarkers [46, 47]. The pathologist must anticipate the IHC and molecular biological tests to be performed by evaluating the size of the sample, the percentage of “viable” tumor cells and the zones of necrosis. Due to the technological progress and in the interest of the well-being of the patients, there is a trend toward small samples, especially given that tumors are detected at earlier stages, are often peripheral and accessible to small caliber flexible endoscopes [48]. The practice of cytological sampling, which is becoming more and more frequent in thoracic oncology, must also be taken into consideration for immunocytochemical and molecular analysis [49–53].

### The tests of choice

The analysis of predictive biomarkers for targeted therapies is performed applying molecular biological and IHC techniques. The detection of genomic alterations is carried out using targeted approaches (sequencing of a single gene or of a few genes) or by NGS or less often by mass array (sequencing of up to several hundred genes using panels of different size). Fluorescent *in situ* hybridization (FISH) techniques can detect certain therapeutic targets (rearrangements in *ALK*, *ROS1*, *RET*, and amplifications in *MET*). Most of the molecular tests are optimally managed by the department/subdivision of molecular pathology. The evaluation of protein expression of certain therapeutic targets (PD-L1, ALK, ROS1) by IHC and FISH analysis are performed by the department of clinical/surgical pathology [54]. The pathologist’s decision to select different tests depends on a number of situational circumstances. Some pathologists may have access to an “in-house” NGS platform with gene panels of varying sizes, including comprehensive genomic profiling panels with over 300 genes. On the other hand, certain institutions choose to outsource molecular biology analyses to academic or commercial laboratories, mainly focusing on NGS tests. Targeted sequencing tests, however, can typically be conducted within the pathology laboratory. The pathologist has to possess a strong expertise not only in morphological analysis, incorporating IHC and molecular testing from targeted sequencing but also in NGS analysis [55]. The pathologist must include in these analyses the initial quality and quantity of the biological material, in particular the percentage of tumor cells, but also the tumor surface and the percentage of necrotic tissue [56, 57]. The objective of the pathologist is to provide a precise histological diagnosis to perform IHC including the analysis of PD-L1 and ALK, and to generate a comprehensive molecular profile by prioritizing NGS over successive sequencing of individual oncogenes, at the time of diagnosis and tumor progression. A major issue of NGS in the health care system could be the cost and the difficulty to get associated funding for testing the majority of NSCLC patients at diagnosis and at tumor progression. However, costs associated with NGS are dramatically decreasing these last few years, while targeted therapy itself represents a major cost driver [58, 59]. Data obtained from NGS not only enables prediction of response to treatment, but also resistance, and could thus prevent administration of unnecessary (and costly) therapies. Whether or not a test can be performed must be the responsibility of the pathologist, who may decide not to send the sample to the molecular biology platform in case of an insufficient amount of tumor cells [57]. The pathologist must then inform the oncologist/other clinicians to perform another tissue biopsy and/or depending on the case and the clinical state of the patient to perform a liquid biopsy.

### Optimizing the management of sample handling for comprehensive reporting

The pathologist’s proficiency in specimens handling is crucial through the entire clinical process, encompassing pre-analytical, analytical, and post-analytical stages, until the results are transmitted to the oncologist. Optimal handling reduces the delay to reporting the results and respects the international recommendations on turn-around times [60]. It is important to report the histological, IHC, and molecular results within a similar time frame. This helps to avoid therapeutic decisions only based on the results of IHC and targeted molecular tests (*EGFR*). It has also become possible to employ ultra-fast NGS [61, 62]. If the molecular tests with tissue and liquid biopsies are performed simultaneously for the same patient, the results must be interpreted at a similar time to avoid initial decisions based only on the results of the liquid biopsy, since the latter is more quickly obtained. This underlines the interest of the pathologist to also develop molecular workflows using liquid biopsies.

### Involvement of the pathologist in the area of liquid biopsies

As mentioned above, the molecular pathologist should ideally integrate the possibility of analyzing molecular targets by assessing liquid biopsies, including blood, pleural, and cerebrospinal fluids into his/her analysis repertoire [63–66]. Investing in the area of liquid biopsies may appear to be a challenge, but harbors the unquestionable advantage of obtaining a global overview of the predictive biomarkers in thoracic oncology by centralizing the tests from a single molecular pathology platform and, in parallel, the morphological and IHC analyses. This allows an optimal summary of the results to be transmitted to the oncologist.

### Understanding the biomarkers of response to targeted therapies and immunotherapies

The landscape of predictive biomarkers in thoracic oncology is constantly changing [18, 19, 67]. This is the consequence of novel discoveries and insights into the biology of lung cancers, of the findings of studies into translational and clinical research and clinical trials. The pathologist is thus required to regularly update her/his knowledge in this area, but also in the areas of molecular biology and novel sequencing technologies and bioinformatics. The pathologist has also the responsibility for training the technicians and residents in pathology in the molecular biology domain and in a continuous manner. Many oncogenic drivers in NSCLC have concurrent mutations and those concurrent mutations may be prognostic or predictive biomarkers with respect to targeted therapies or immunotherapies. However, given the rarity of many genomic alteration in NSCLC (as for example *BRAFV600E*, *HER2*, *ROS1*, *NTRK*) and the incidence of each of the concurrent mutations, a registry would be strongly advisable as the best method of developing larger cohorts of patients to further investigate the role of concurrent mutations. In this context, we have to keep in mind the critical role of the pathologists in translational research programs thanks to their specific expertise and their knowledge both in morphology and in molecular biology as well as their pivotal involvement in biobanking activities [68].

### Novel issues in the treatment of early stage non-small cell lung cancers

The recent approval of the administration of osimertinib after complete surgical resection of IB-IIIA stage NS-NSCLC presenting with an actionable mutation in *EGFR* (L858R mutation and exon 19 deletion) based on the results of the ADAURA trial calls for the evaluation of *EGFR* mutations in early-stage NSCLC [69]. Therefore, the pathologist must adopt these tests in daily practice [22]. Furthermore, promising results of phase III clinical trials on neoadjuvant immuno-chemotherapy require evaluation of *EGFR* and *ALK* status a potential negative stratifier using preoperative biopsies [70, 71]. The effect of these neoadjuvant treatments on the proliferation of the tumor must be evaluated on surgical specimens by estimating the percentage of residual tumor cells in relation to the tumor bed [72]. Two indicators must be reported: the absence of residual tumor cells is an indicator of a complete pathological response (“pCR”) and the persistence of less than 10% tumor cells define major pathological response (“MPR”) [72]. These criteria of evaluation of surgical specimens require expertise. Training programs and the setup of validated inter-laboratory tests with quality controls are warranted. In general, the socio-economic issues and the impact of these peri-operative treatments on public health are crucial, since they lead to prolonged follow-up of patients and avoid tumor recurrence, the health care of which is particularly costly [73].

## Challenges faced by the thoracic pathologist and suggestions for optimization

When deciding on the treatment of patients with NSCLC, regardless of the stage or histological type, predictive biomarkers for approved targeted therapies or immunotherapies, must be taken into account, prior considering chemotherapy and/or radiotherapy where appropriate. The list of these biomarkers is becoming increasingly longer, which requires changes to the daily practice of pathologists and constant reconsideration of adding certain analyses to the workflow [67]. The pathologist must keep up to date with the international recommendations, new classifications, the tumor, node, metastases (TNM) classification, and novel morpho-molecular elements [74]. The training and education of pathologists in molecular pathology should aim to optimize both their theoretical knowledge and practical skills in these aspects [75].

Several recent developments should improve and facilitate the daily work of the molecular pathology laboratory. The developments in digital pathology could be integrated into the evaluation of the pre-analytical phase of molecular pathology, with control of the quality of the sample (quantification of the percentage of tumor cells and areas of necrosis) before the sample is sent to the molecular biology platform (https://www.univ-cotedazur.eu/msc/european-msc-molecularpathology) [76–78]. Digital pathology should help reduce the delay in obtaining results and thus accelerate the therapeutic decision making (https://www.univ-cotedazur.eu/msc/european-msc-molecularpathology) [76, 79]. However, the developments in digital pathology may encounter several problems, depending on the institution and laboratories, notably with respect to different interfaces of information systems, the software and the work of the laboratory technicians and the pathologist. An increase in the automation of procedures including nucleic acid extraction (from tissue or liquids), quality control, the preparation of libraries and sequencing, and bioinformatic tools should allow in-house adoption of more and more complex genomic analyses [80]. The pathologist must be one of the essential players in therapeutic decision making in multidisciplinary boards [11]. However, the regular participation at molecular boards is time consuming and requires the pathologist to reorganize the workload. Another challenge concerns the setup of reflex NGS, without waiting for the request from the clinician, in particular for advanced staged tumors [80]. Managing the impact on laboratory staff and costs also needs to be addressed, as well as the storage, analysis, and application of complex data, including also data protection [80, 81]. However, this new practice requires new competences. Finally, the molecular pathologist also has to deal with challenges addressed by new European regulations on the performance of biological tests (In Vitro Diagnostic Regulation or IVDR), regulations that will certainly modify the daily practices, particularly of molecular pathologists [82].

## Short- and mid-term perspectives

With the constant new discoveries in the pathophysiology of NSCLC and the emergence of new therapeutic molecules, the thoracic pathologist is faced with many prospects in the field of molecular pathology (Table 3)

**Table 3:** Perspectives for in-house molecular pathology testing in thoracic oncology

NGS using CGP panels
CGP panels for NGS from liquid biopsies and tissue biopsies at both initial diagnosis and tumor progression
Reflex testing using CGP panels for any histological subtypes of NSCLC
Combined assessment of somatic and germline genomic alterations in thoracic oncology for better decision making for precision oncology (prediction of primary drug resistance; prediction of drug toxicity)
Novel bioinformatic tools for easier assessment and shorter turnaround time for transmission of reports
Expanding indications for liquid biopsies in thoracic oncology
Detection of minimal residual disease in early stage NSCLC in daily practice
Integration of a liquid biopsy as a screening tool for detection of lung cancer
Development of liquid biopsy testing for predictive biomarkers of ICIs efficiency
Adopt the in vitro diagnostics/devices regulations for in-house molecular testing
Develop diagnostic tests with liquid biopsies as complimentary to testing with tissue biopsies for diagnosis
Harmonize NGS use with liquid biopsies avoiding research use only testing and leading to universal use of CE-IVD testing
Consider different biopsy sources as a pivotal tool for the concept of integrative pathology
Liquid biopsies (blood, pleural fluid and pericadial effusion, cerebrospinal fluid) combined with tissue biopsies for diagnosis, prognosis, and predictive biomarker evaluation in thoracic oncology
Integration of digital pathology and artificial intelligence algorithms from hematoxylin and eosin stained tissue sections for genomic alterations detection
Combination of complex in situ multiplex analyses and genomic alterations profiles for prediction of targeted therapies and/or immunotherapies responses, toxicities, and/or resistances

*CGP* comprehensive genomic profiling, *NSCLC* non-small cell lung cancer, *ICIs* immune check point inhibitors, *NGS* next-generation sequencing

### The use of comprehensive genomic profiling and RNA sequencing technologies

The size of the gene panels used for NGS varies within the laboratories and depends on the request (from less than 15 to more than 500 genes) [83–85]. With adaptations, these panels can be used for the examination of tissue or liquid samples. The large panels, or CGP (comprehensive genomic profiling) panels, will probably soon be increasingly used in thoracic oncology. These panels provide comprehensive genomic information, including complex marker like TMB, MSI, or HRD, and allow the patients to be included into clinical trials after decisions are made by a committee during multidisciplinary consultation meetings or MTB [11]. However, several issues still limit the use of these large panels and favor the choice of alternative panels of a small or medium size: (i) the limited access to sequencing equipment, in particular for DNA extracted from plasma, which requires a greater level of sensitivity when using large panels, (ii) the tumor cell content and quantity and quality of the extracted nucleic acid, which can limit the sensitivity of the analyses, (iii) the turnaround time to obtain results, (iv) the decision to internalize or externalize analyses (“in-house” versus “outsourced testing”) that require substantial expertise in bioinformatics, and (v) the cost and reimbursement of analyses. In the future, sequencing of the entire exome or genome in combination with the transcriptome will expand, raising further challenges in terms of analyses, turnaround time, data storage capacity, and the expertise of the molecular pathologist [84, 85]. The genomic signatures will likely become increasingly complex to interpret, resulting in new classifications [86–91]. In this context we need to keep in mind that RNA-based NGS has also emerged as a more clinically sensitive approach than DNA-based NGS for the detection of both fusions and splice variants [15]. One major challenge with DNA detection of gene rearrangements is the greater wet laboratory and bioinformatics pipeline complexity to accurately detect the different breakpoints. Moreover, RNA NGS can confirm canonical fusions in samples that could not be defined definitively as actionable fusions based on DNA NGS because the fusions can have uncommon partner genes but also break-points in intergenic regions [15]. Finally, with DNA sequencing, there is also a risk of reporting rearrangements that do not encode clinically actionable fusion products [15]. The molecular pathologist will therefore need to be adept even further at handling and understanding complex multi-level data [89–91].

### Artificial intelligence and molecular pathology

As mentioned above, digital pathology will certainly and progressively enhance or replace the present practices [77, 78, 92–95]. In addition to improving the diagnostic processes, digital pathology can compile many information and imaging data. All of this leads to the consideration of using artificial intelligence (AI) not only to support for the morphological diagnosis, but also to identify different prognostic and predictive biomarkers [94, 95]. The improvement of these algorithms will provide a map of increasingly complex morpho-molecular analyses.

### Anticipate primary and secondary resistances to targeted therapies and immunotherapies, as well as their toxicities

Many mechanisms of resistance to personalized treatments can emerge and can be present before starting treatment or at tumor progression in treated patients [36, 37, 96, 97]. Anticipating the different mechanisms of resistance to targeted therapies and immunotherapies will become a major issue, which will avoid administering costly and ineffective medication and will be important to determine the sequence of different targeted therapies for the same or additional targets. Molecular pathology may also participate in evaluating the potential toxicity of certain molecules, notably by integrating the genetic susceptibility of the patients and some polymorphisms. Thus, germline analysis should be systematically combined with somatic alterations of the tumor genome [98].

### Integrative pathology and omics (genome, epigenome, pharmaco-genome, transcriptome, proteome, and exposome)

The information and data of a patient and the tumor are obtained from various sources. Those are primarily clinical data and the results of biological analyses, some of which are already used to evaluate the response to certain therapeutics [neutrophil to lymphocyte (N/L) ratio]. But these data can also be derived from different omic analyses obtained from the genome, epigenome, transcriptome, and proteome [94, 95]. Other data are derived from the pharmaco-genome and exposome that integrate all the information needed for therapeutic decision-making. The technological advances such as spatial transcriptomics and *in situ* multiplex analyses could be used in the future to optimize therapeutic decisions [99–104]. This integrative pathology will be based on the emergence of new skills and professions in the domain of oncology, leading to the onset of next generation pathologists.

### The detection of minimal residual disease (MRD)

One of the major concerns in thoracic oncology is post-operative relapse of lung cancer and how to improve the survival of operated patients. With this aim in view, the minimal residual disease (MRD) analysis will enable new therapeutic strategies to improve patient survival by assessing biomarkers circulating in the blood that can be used for early detection of tumor progression or associated with a poor prognosis [105, 106].

### The emergence of novel predictive biomarkers in immuno-oncology

The development of novel predictive biomarkers in the field of immuno-oncology is a major issue in thoracic pathology, knowing the limit of PD-L1 IHC and even more of the TMB [107–109]. It is probable that the combination of molecular analyses based on genetic alterations and complex analyses of the microenvironment will enable the identification of novel biomarkers predicting the efficacy of immunotherapies. The pathologists have an important role for evaluating these new biomarkers, their location and/or association and their quantification [104, 110]. Finally, the pathologists are strongly involved in different research activities leading to the discovery of new target molecules in tumor and/or microenvironment cells [104, 110].

## Conclusion

**Fig. 2:**
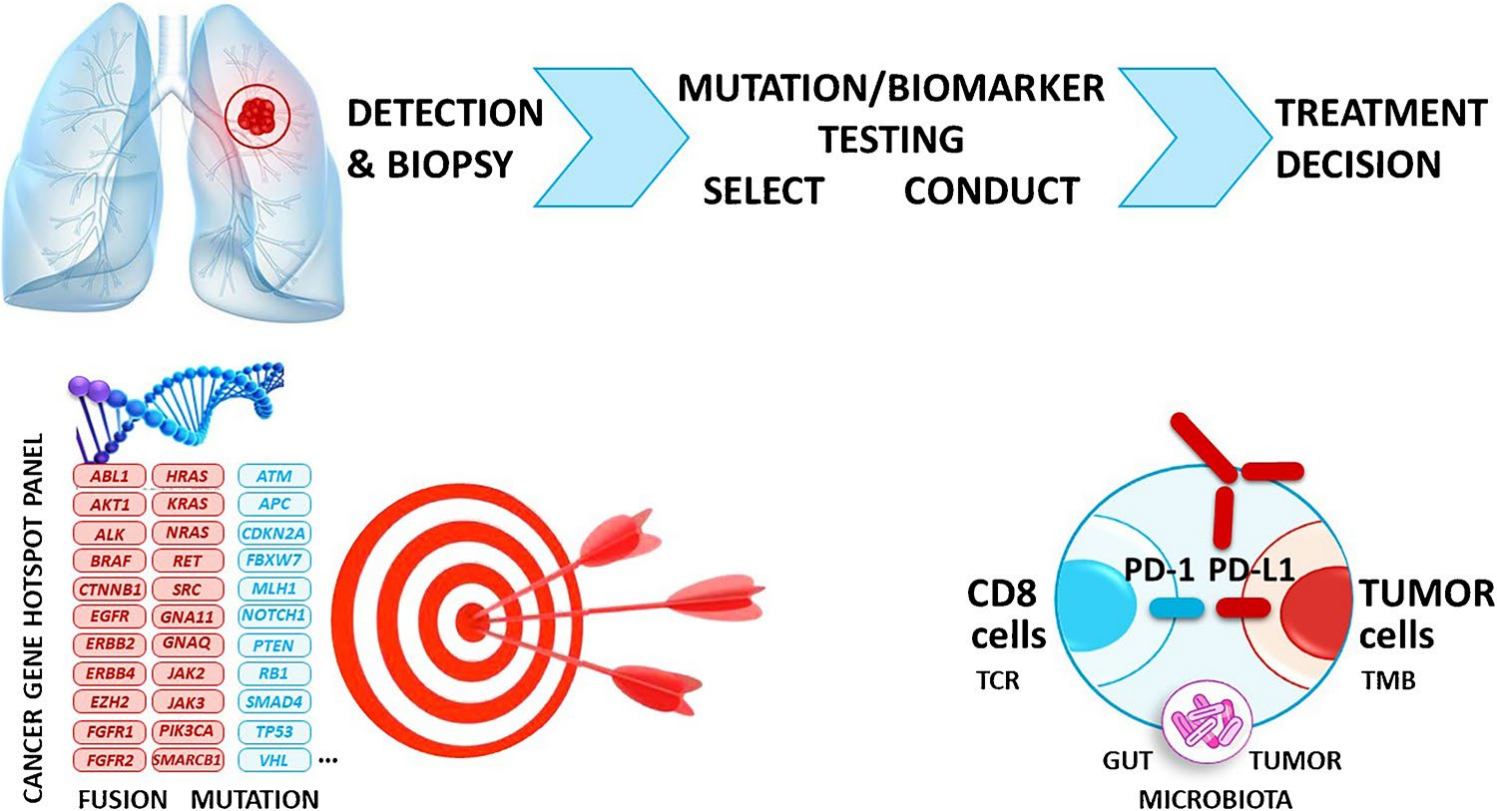
Combined biomarkers for targeted therapy and immunotherapy in thoracic oncology

Despite advances in precision oncology, and especially for thoracic cancer, there are still challenges in the development of molecular biology for lung cancer patients, notably in many European countries. Access to technology and expertise, as well as the cost and reimbursement policies of analyses are important hurdles that must be overcome in this regard [111, 112]. Changes in clinical practice, such as the integration of NGS for the assessment of both tissue and liquid biopsies, but also by combining biomarkers for targeted therapy and immunotherapy should facilitate more comprehensive and faster patient stratification, to endeavor to ensure that no lung cancer patient is left behind (Figure 2). Advances in technology, such as NGS, but also digital PCR, digital pathology, computational pathology, and AI, should contribute substantially to provide faster and more accurate test results. However, it is still important to identify and overcome a few challenges, such as poorly controlled pre-analytical procedures that lead to suboptimal biomarker testing. In this context, new workflows and/or the adaptation of existing laboratory processes can be necessary to set up and implement automation of the different steps, new testing strategies, algorithms, and technologies. As already mentioned, the role of the pathologist in molecular pathology is pivotal and this role will be certainly even more important in the future [11, 55, 56]. However, the thoracic pathologists will face many new challenges in the field of molecular pathology and in precision oncology [90, 113]. Developing educational and outstanding training programs in centers of expertise are nowadays mandatory and an urgent need in order to foster the next generation of thoracic pathologists. Therefore, setting up specific long-term programs in molecular pathology allowing both theoretical and practical knowledge in molecular testing and analyses are certainly an urgent need for the future pathologists [75]. Finally, these programs have to integrate different aspects of the tumor biology, including the genomic and epigenomic, but also the transcriptomic and the proteomic (including *in situ* multiplex analyses), as well as new perspectives in thoracic oncology, notably the complementary approaches brought by liquid biopsy development.
